# Approaches to carrier testing and results disclosure in translational genomics research: The clinical sequencing exploratory research consortium experience

**DOI:** 10.1002/mgg3.453

**Published:** 2018-08-21

**Authors:** Kathryn M. Porter, Tia L. Kauffman, Barbara A. Koenig, Katie L. Lewis, Heidi L. Rehm, Carolyn Sue Richards, Natasha T. Strande, Holly K. Tabor, Susan M. Wolf, Yaping Yang, Laura M. Amendola, Danielle R. Azzariti, Jonathan S. Berg, Katie Bergstrom, Leslie G. Biesecker, Sawona Biswas, Kevin M. Bowling, Wendy K. Chung, Ellen W. Clayton, Laura K. Conlin, Gregory M. Cooper, Matthew C. Dulik, Levi A. Garraway, Arezou A. Ghazani, Robert C. Green, Susan M. Hiatt, Seema M. Jamal, Gail P. Jarvik, Katrina A. B. Goddard, Benjamin S. Wilfond, The members of the CSER Actionability and Return of Results Working Group

**Affiliations:** ^1^ Treuman Katz Center for Pediatric Bioethics Seattle Children's Research Institute Seattle Washington; ^2^ Center for Health Research Kaiser Permanente Northwest Portland Oregon; ^3^ Institute for Health and Aging University of California San Francisco California; ^4^ Medical Genomics and Metabolic Genetics Branch of the National Human Genome Research Institute Bethesda Maryland; ^5^ Broad Institute of MIT and Harvard Cambridge Massachusetts; ^6^ Harvard Medical School Boston Massachusetts; ^7^ Partners Personalized Medicine Boston Massachusetts; ^8^ Laboratory for Molecular Medicine Partners Healthcare Personalized Medicine Cambridge Massachusetts; ^9^ Knight Diagnostic Laboratories and Department of Molecular and Medical Genetics Oregon Health and Science University Portland Oregon; ^10^ Department of Genetics The University of North Carolina at Chapel Hill Chapel Hill North Carolina; ^11^ Stanford Center for Biomedical Ethics Palo Alto California; ^12^ University of Minnesota Law School Medical School and Consortium on Law and Values in Health, Environment & the Life Sciences Minneapolis Minnesota; ^13^ Department of Molecular and Human Genetics Baylor College of Medicine Houston Texas; ^14^ Department of Medicine Division of Medical Genetics University of Washington Seattle Washington; ^15^ Texas Children's Cancer Center and the Department of Pediatrics Texas Children's Hospital Baylor College of Medicine Houston Texas; ^16^ Department of Pediatrics The Children's Hospital of Philadelphia Philadelphia Pennsylvania; ^17^ Hudson Alpha Institute for Biotechnology Huntsville Alabama; ^18^ Department of Pediatrics Columbia University New York New York; ^19^ Department of Medicine Columbia University Medical Center New York New York; ^20^ Center for Biomedical Ethics and Society Vanderbilt University Medical Center Nashville Tennessee; ^21^ Division of Genomic Diagnostics The Children's Hospital of Philadelphia Philadelphia Pennsylvania; ^22^ Eli Lilly and Company Indianapolis Indiana; ^23^ Department of Medical Oncology and Center for Cancer Precision Medicine Dana‐Farber Cancer Institute Boston Massachusetts; ^24^ Department of Medicine Brigham and Women's Hospital Boston Massachusetts; ^25^ Division of Genetics Department of Medicine Brigham and Women's Hospital Boston Massachusetts; ^26^ Department of Molecular Genetics University of Toronto Toronto Ontario; ^27^ Department of Genome Sciences University of Washington Seattle Washington; ^28^ Department of Pediatrics University of Washington School of Medicine Seattle Washington

**Keywords:** carrier testing, exome, genome, secondary findings, translational genomics research

## Abstract

**Background:**

Clinical genome and exome sequencing (CGES) is primarily used to address specific clinical concerns by detecting risk of future disease, clarifying diagnosis, or directing treatment. Additionally, CGES makes possible the disclosure of autosomal recessive and X‐linked carrier results as additional secondary findings, and research about the impact of carrier results disclosure in this context is needed.

**Methods:**

Representatives from 11 projects in the clinical sequencing exploratory research (CSER) consortium collected data from their projects using a structured survey. The survey focused on project characteristics, which variants were offered and/or disclosed to participants as carrier results, methods for carrier results disclosure, and project‐specific outcomes. We recorded quantitative responses and report descriptive statistics with the aim of describing the variability in approaches to disclosing carrier results in translational genomics research projects.

**Results:**

The proportion of participants with carrier results was related to the number of genes included, ranging from 3% (three genes) to 92% (4,600 genes). Between one and seven results were disclosed to those participants who received any positive result. Most projects offered participants choices about whether to receive some or all of the carrier results. There were a range of approaches to communicate results, and many projects used separate approaches for disclosing positive and negative results.

**Conclusion:**

Future translational genomics research projects will need to make decisions regarding whether and how to disclose carrier results. The CSER consortium experience identifies approaches that balance potential participant interest while limiting impact on project resources.

## 1 INTRODUCTION

Clinical genome and exome sequencing (CGES) is primarily used to address specific clinical concerns by detecting risk of future disease, clarifying diagnosis, or directing treatment (Biesecker & Green, 2014). The American College of Medical Genetics and Genomics (ACMG) currently recommends offering secondary results for 59 genes for which medical actions that can ameliorate or prevent harm are available (Kalia et al., 2017). Despite the clinical availability of CGES, there remains a need for translational research to better understand the benefits and limitations that can guide clinical use and policy. In addition, CGES makes possible the disclosure of autosomal recessive and X‐linked carrier results as additional secondary findings, and research about the impact of carrier results disclosure in CGES is complicated and timely. None of the secondary results recommended by the ACMG are carrier results for autosomal recessive disorders (Green et al., 2013; Kalia et al., 2017). Carrier testing may be perceived as valuable for those seeking to assess risks for a current or future pregnancy (Edwards et al., 2015), but when carrier results are offered as a secondary finding in CGES, the level of interest and impact may vary widely.

The disclosure of carrier status as part of CGES is complicated because offering results for hundreds of conditions is in stark contrast to the standard of focused carrier testing over the last 40 years. Carrier testing has expanded slowly since the 1970s with only a handful of conditions recommended by professional guidelines from the American College of Obstetrics and Gynecology (ACOG) and the ACMG (ACOG Committee on Genetics 2004; American College of Obstetricians and Gynecologists Committee on Genetics 2011; Kalia et al., 2017). Gradual expansion has occurred following health services research suggesting that benefits to carrier testing exist and the risks are modest (Grody, 2016). Yet even for the widely available cystic fibrosis carrier testing, it is not clear how often testing is offered to patients and how often tests are performed (Ioannou et al., 2014). Numerous factors may have contributed to the relatively slow expansion. One is the belief that only serious disorders should be included, complicated by lack of consensus on the threshold for seriousness (Wertz & Knoppers, 2002). Furthermore, there is a concern that carrier testing and prenatal screening could devalue the lives of those with disabilities by allowing a single feature to represent the entire individual (Parens & Asch, 1999; Parens & Asch, 2000). Finally, some worry that such information may create difficult decisions for patients or family members (Press, Wilfond, Murray, & Burke, 2011).

These concerns, still unresolved, have new urgency as technologies make it possible to examine carrier status for thousands of genes. Currently available commercial panels can detect variants for hundreds of rare conditions (Lazarin et al., 2013; Nazareth, Lazarin, & Goldberg, 2015). Yet it is not clear if the identification and disclosure of carrier status is an appropriate use of CGES technology, which has the potential to provide information on nearly any condition with recessive or X‐linked inheritance. There is currently no consensus regarding how to approach carrier testing and results disclosure from CGES. There are limited data on the risks and benefits of expanded carrier results disclosure and limited translational genomics research that evaluates carrier screening using CGES (Kauffman et al., 2017).

The clinical sequencing exploratory research (CSER) consortium, funded by NHGRI and NCI from 2011 to 2017, was made up of 18 extramural research projects and one NHGRI intramural project (Green et al., 2016). The goals of these projects were to understand the clinical implications of CGES and create an evidence base to support appropriate clinical integration of this emerging technology. Many CSER translational research projects disclosed carrier results as a secondary result. Several projects have published outcomes that include carrier testing (Biesecker et al., 2018; Cirino et al., 2017; Lewis et al., 2018; Parsons et al., 2016; Vassy et al., 2017; Wynn et al., 2018). The Consortium's Actionability/Return of Results Working Group sought to understand the variability in CSER approaches to identifying and disclosing carrier results, including (1) selection of genes and variants; (2) choices given to research participants regarding which results to disclose; (3) results communication approaches; and (4) project outcomes, including the proportion of individuals with carrier results and which genetic variants are commonly identified. The broad range of experiences across CSER projects allowed us to distill key considerations important for guiding future translational genomics research projects that will inform decisions regarding whether and how to disclose carrier results. This research may in turn help to guide policy decisions about clinical services.

## 2 MATERIALS AND METHODS

A CSER working group requested that the 19 CSER research projects, including U‐award projects, R‐award projects, and the NIH project, indicate which projects offer and/or disclose carrier results. The U‐award projects and the NIH project assessed sequencing results in the healthcare setting. Eight of the nine U‐award projects and the NIH project disclosed carrier results. The R‐award projects were heterogeneous in design regarding research results. Only three R‐award projects disclosed any results, and two included carrier results. Thus, 13 projects were identified as disclosing results, 11 of which included carrier results. In order to ensure that the questions were relevant for each project, we developed a survey in collaboration with representatives from each of these 11 projects.

The quantitative, descriptive survey gathered information on: project characteristics, how many carrier status genes/conditions were screened for, which pathogenicity categories were disclosed for carrier results and other secondary findings (findings unrelated to the primary indication for the test), how results were disclosed and by whom, and project‐specific outcomes regarding the number of participants, which genes (for carrier variants) were identified, and whether the participants chose to receive carrier results. Once finalized, the survey was distributed to each project's principal investigator or designee. We conducted a phone interview for the representative and interviewer (KMP) to review the survey questions. Responses were entered electronically by the interviewer using REDCap.

Initial data collection took place between March and August 2015. Follow‐up correspondence and conversations took place through February 2016. A final follow‐up conversation occurred between May and August 2016 to gather updated data (current for each site as of May 1, 2016). We utilized descriptive statistics because the survey was administered to only 11 projects.

## 3 RESULTS

### 3.1 Study characteristics and background

Of the 11 project teams that completed the survey, two performed genome sequencing only, eight performed exome sequencing only, and one performed both. The mix of adult and child participants varied across the projects and included those that focused exclusively on the pediatric population, those with a mix of pediatric and adult participants, and those primarily serving adults. Specific clinical problems targeted by the projects included cardiovascular diseases (*n* = 6), cancer (*n* = 5), and birth defects and developmental delay (*n* = 4). Three projects recruited a healthy/general population and one focused on preconception carrier screening. More detailed information about the research projects has been previously published in an overview of the CSER consortium (Green et al., 2016).

### 3.2 Selection of genes/conditions to disclose for carrier status

As illustrated in Table 1, projects disclosed carrier results for as few as three and as many as 4600 autosomal recessive or X‐linked genes. Six projects developed a gene list prior to sequencing and disclosed only carrier status variants in the genes on the list. In contrast, four projects utilized a postsequencing review process, deciding after analysis of each participant's exome/genome whether to disclose the observed variants based on evidence review of the relevant genes and variants. One project utilized both methods, automatically disclosing carrier results for three genes but deciding after analysis whether to disclose other gene variants.

**Table 1:** Number of genes or conditions and timing of review for carrier status disclosure among CSER projects

Project	Presequencing	Postsequencing
Dana‐Farber Cancer Institute	3 (genes)	
HudsonAlpha^a^	3 (genes)	OMIM recessive disorders (conditions)
University of Washington	10 (genes)	
Seattle Children's Hospital	29 (genes)	
Kaiser Permanente Northwest	668 (genes)	
Children's Hospital of Philadelphia	186 (genes)	
Columbia University		OMIM recessive disorders (conditions)
University of North Carolina, Chapel Hill		OMIM recessive disorders (conditions)
Baylor College of Medicine		OMIM recessive disorders (conditions)
National Human Genome Research Institute		1508 (genes)
Brigham and Women's Hospital		4600 (genes)

a Always offers to disclose carrier status for three genes (*CFTR*,* HBB*,* HEXA*), but also performs trio sequencing so will offer to disclose carrier status for disease‐linked genes in OMIM when both mother and father are carriers.

**Figure 1:**
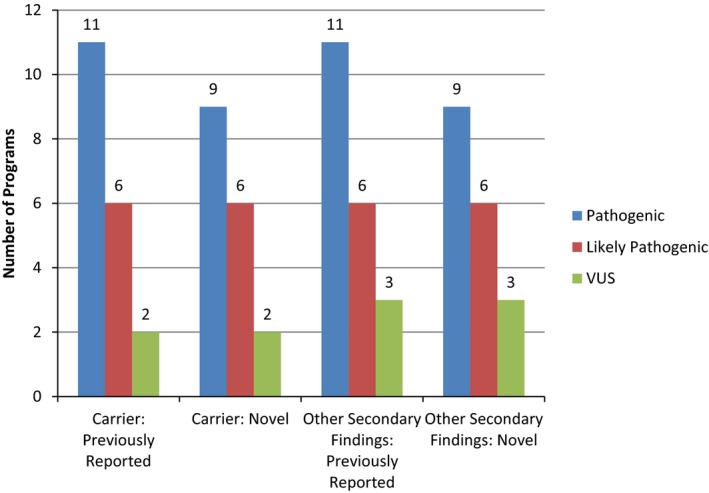
Disclosure of pathogenicity variant categories for carrier and secondary findings in CSER projects

Figure 1 illustrates the variant pathogenicity classes disclosed by each project. All projects disclosed carrier results for previously reported variants with sufficient evidence to be classified as pathogenic, while nine disclosed carrier results for novel variants that were classified as pathogenic. Six projects disclosed carrier results for likely pathogenic variants (both previously reported and novel). Only two projects disclosed carrier results for variants of uncertain significance (VUS) (both previously reported and novel). One of these two projects reported only a subset of VUS, those variants that favor pathogenic, which accounted for <1% of the VUS results they saw. For comparison, projects treated the disclosure of other secondary findings (e.g., variants in potentially actionable autosomal dominant genes) similarly, the one exception being that three projects disclosed VUS (both previously reported and novel) for these other secondary findings.

### 3.3 Participant choices

Nine projects allowed participants to choose whether or not to receive some or all of the available carrier results, while the remaining two required disclosure as part of the study design. Of the projects disclosing carrier results optionally (i.e., offering), all but one reported between 85%–100% of participants being willing to receive some or all carrier results, while the remaining project reported only 27% uptake. This latter project used a study design in which the primary diagnostic results and medically actionable secondary results were disclosed first. Participants were then asked to make decisions about other categories of results (e.g., carrier status, pharmacogenomics, *APOE* status). Those who desired the further results were asked to call to request this analysis. Notably, among those who chose to learn any of their additional results in this project, 88% opted for carrier results, suggesting that the lower observed uptake was likely not specific to carrier status but rather a lower uptake of additional genomic results in general.

### 3.4 Results communication

Six projects always communicated positive carrier results in person (four by a genetic professional, one by a nongenetic professional, and one by both). Four projects sometimes communicated positive results in person (two by a genetic professional, two by both), using a phone call as the alternative to an in‐person meeting. Two projects also used some other electronic method (i.e., website) as an alternative. Some projects (*n* = 3) provided a letter/email as a follow‐up or in addition to an in‐person discussion. One project disclosed carrier results to physicians rather than directly to participants.

Only six of the eleven projects communicated negative carrier results. Three of these always communicated the negative carrier results during the course of an in‐person meeting (one by a genetic professional, one by a nongenetic professional, and one by both). This could be due to study design rather than an explicit decision to present negative results in this format (e.g., an in‐person meeting was already taking place and therefore was a logical time to disclose negative results). One of the six projects sometimes disclosed negative carrier results in person, using a phone call as the alternative. The final two projects never disclosed negative carrier results in person and instead sent a letter/email to inform the participant. Some of the projects that conveyed negative results in person also provided the participant with a letter or other document that summarized the findings.

### 3.5 Outcomes

**Figure 2:**
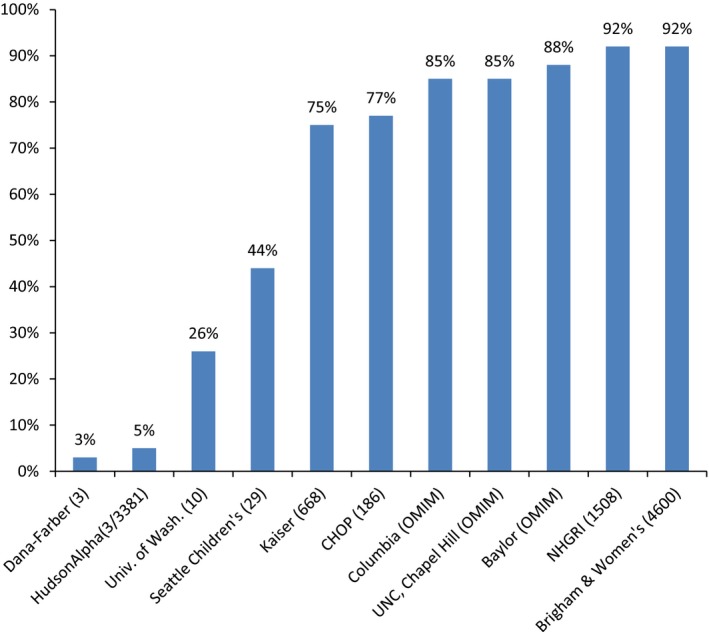
Participants with at least one positive carrier result disclosed in CSER projects. This figure includes the total number of conditions reviewed by each project as indicated in Table 1.

The number of participants in each CSER project that completed sequencing and had carrier results analyzed ranged from 19 to 640. The percentage of participants who received at least one carrier result ranged from 3% to 92% per project, with variation primarily due to differences in the number of genes for which variants were reviewed (see Figure 2). Between one and seven results were disclosed to those participants who received any positive result.

Table 2 shows the genes in which variants were most frequently disclosed in each project. Variants in these genes cause some of the most common recessive disorders in the U.S. population (Adam et al., 2016).

**Table 2:** Most commonly disclosed genes for carrier status per CSER project^a^

Gene (condition)	Number of projects that list gene among the three most commonly disclosed	Number of projects that would disclose	Disease prevalence
*HFE* (*HFE*‐associated hereditary hemochromatosis)	5	7	1/200–1/400
*GJB2* (nonsyndromic hearing loss)	5	9	1/7,000
*CFTR* (cystic fibrosis)	4	11	1/3,200 (Northern European Ancestry)
*BTD* (biotinidase deficiency)	4	8	1/60,000
*SERPINA1* (alpha‐1 antitrypsin deficiency)	4	8	1/50,00–1/7,000
*HBB* (sickle cell disease)	2	11	1/300–1/500 (African American Ancestry)

a Genes reported by only one of the 11 CSER projects as most frequently disclosed include *HEXA*,* ABCA4*,* CYP21A2*,* MUTYH*,* LRTOMT*,* F11*,* IDUA*,* ACADM*,* CD36*,* DUOX2*,* OTOF*,* ABCC6*,* C2*,* SACS*, and *SMPD1*.

### 3.6 Carrier results as the primary finding

In contrast to the other 10 projects for which carrier results were secondary findings, carrier results were the primary finding for one project that was focused on preconception carrier screening. This project performed genome sequencing and included only adult participants who were interested in preconception carrier screening. The study disclosed results for 668 genes (corresponding to 728 gene‐condition pairs) and utilized a presequencing review process, developing a gene list prior to sequencing and disclosing only carrier status variants in the genes on the list. This project disclosed carrier results for both previously reported and novel variants with sufficient evidence to be classified as pathogenic or likely pathogenic. Due to the goals of the study, carrier result reporting was required for all participants. Positive results were always communicated in person by a genetic professional while negative results were reported via letter/email. As of May 1, 2016, 135 participants completed sequencing and had carrier results analyzed. 75% of these participants received at least one carrier result.

## 4 DISCUSSION

CSER project approaches to disclosing carrier results varied, including whether carrier results were the primary or secondary finding, the number of genes reviewed, the variant categories of pathogenicity that projects disclosed, and the results communication approach. While this descriptive study does not provide sufficient evidence to determine the best approach to disclosing secondary carrier results from CGES, we identify some specific issues that translational genomics research projects face and ways in which CSER projects approached them. For example, several projects returned carrier results for *HFE*‐associated hereditary hemochromatosis but it was not within the scope of this survey to determine why that decision was made by each individual project. While these results would not typically be disclosed in the clinical setting, research projects have greater flexibility related to their scientific goals (Jarvik et al., 2014).

Most participants in these translational genomics research projects were willing to receive their carrier results. Ascertainment of carrier status in an adult has the ability to inform reproductive decision‐making, facilitate early diagnosis and treatment for their child with a genetic condition, and alert other family members to their risk status (Grody et al., 2013; Himes et al., 2017 Jul; Schneider et al., 2016). Participants may value this information for reasons including, but not limited to, increasing sense of control, lessening anxiety, helping with decision‐making, and preparing for the possibility of a child with the condition (Schneider et al., 2016). However, some participants may not want this information if they are not of reproductive age or if the information is not likely to change reproductive decisions or might produce stress (Schneider et al., 2016). Because reproductive decisions involve personal values, individuals will vary in their attitudes toward, and use of, carrier status information, but some participants endorse the value of choice, including the choice of whether or not to receive carrier status results (Schneider et al., 2016).

More translational genomics research is needed on the implementation of CGES for ascertainment of carrier status. As the CSER projects illustrate, although some projects focus primarily on carrier results, most CGES projects face the question of whether to include carrier results among the secondary findings they offer to disclose. As commentators have suggested, translational research projects do not have a compelling obligation to disclose carrier results, and instead may decide whether or not to offer such results to participants (Fabsitz et al., 2010; Jarvik et al., 2014; Presidential Commission for the Study of Bioethical Issues 2013; Wolf et al., 2012). However, given the high percentage of CSER participants who were willing to receive carrier results, future translational genomics researchers should consider the possibility, both to study the utility of offering these results and to respond to participants’ interest (Ravitsky & Wilfond, 2006; Richardson & Belsky, 2004). When carrier results disclosure is not a scientific aim of a research project, researchers need to balance offering these results with obligations to steward resources to address research questions when deciding whether to offer results. Results disclosure should meet CLIA standards for analytic validity, and ensure appropriate results interpretation and communication by adequately trained personnel.

Based on our experience, we identify some approaches that consider participant interest while bearing in mind the impact on project resources. First, a project can limit the number of genes or conditions included for carrier variant reporting and disclose results based on frequency and seriousness, thereby focusing on the most clinically meaningful results to provide the greatest value given limits of capacity (see Figure 2). Second, a project can limit disclosure to a predetermined list of variants, thereby reducing time spent on pathogenicity classification and orthogonal validation. Third, a project can decide to disclose only pathogenic variants. Fourth, a project can disclose results only when both partners planning a pregnancy are carriers for variants in the same gene; this would lead to a dramatic reduction in the number of positive results disclosed and focus on information that would impact clinical management (Wald, George, Wald, & Mackenzie, 1993). Finally, projects can ask participants about carrier results analysis following targeted deliberation (e.g., using a specific decision aid or being asked to call, text, or register online to request this analysis separate from the primary decision to enroll in the study and receive primary results). The CSER project that utilized such an approach experienced a much lower percentage of participants who ultimately opted to receive carrier results, thus limiting the effort spent by the research team on interpretation, while still providing carrier results for interested individuals.

### 4.1 Limitations

This study has a few limitations. First, the CSER consortium was not designed for direct comparisons of various approaches across projects. Carrier detection was the primary goal of the sequencing for only one of the 11 projects; other projects might have used different approaches if carrier screening was a primary goal. While including one study primarily aimed at carrier screening with 10 studies for which carrier results were secondary might bias our findings because of a specific study population or a more deliberative process in that study, our findings show that this one carrier‐focused study did not differ from the others with respect to interest in carrier screening or percentage of participants with positive carrier results. Our analysis was limited to projects using CGES and did not include other platforms such as expanded carrier screening panels using targeted genotyping or sequencing. The high uptake for carrier status results observed in most projects may have reflected the distinctive populations of individuals who were willing to receive clinical sequencing at this stage of its development and more diverse populations and settings might show a different frequency of uptake. Additionally, we recognize the importance of data about the impact of carrier results disclosure on participants but herein we limit our focus to the variability of approaches taken by the various CSER projects. Nonetheless, this is the largest description of approaches to carrier results disclosure in translational genomics sequencing research to date. This description is valuable because multiple projects are represented with distinct study designs allowing insight into the breadth of approaches that were used in the CSER consortium.

### 4.2 Considerations for future translational genomics research

Based on the CSER experience, we are able to provide insight into research approaches using CGES for carrier status and provide suggestions for future translational genomics research that evaluates the clinical impact of disclosing carrier results in CGES. Carrier results may be offered to research participants either because this disclosure addresses specific research questions or because the research team is motivated by the potential personal value of this information to participants to justify disclosure as secondary results. The following considerations are focused on disclosure of these secondary results because of potential value to participants and not related to addressing specific research questions, which may direct the specific approach. Each consideration leads to specific questions that would need to be answered by future projects, as seen in Table 3. These considerations are based in part on the findings from this study but also on our experiences as researchers in the field.

**Table 3:** Four challenges to address in considering the use of CGES to ascertain and disclose carrier results in translational genomics research

General challenges	Specific questions
Whether or not to offer to disclose carrier results	What is the likely perceived utility of carrier results in this study population? Can the research team inform their decisions on whether and how to offer carrier status findings through pilot studies or other research on participant interest in these findings and the feasibility of disclosure?
Selection of genes/conditions to disclose for carrier status	What specific criteria will be used? Will the genes/conditions selected for disclosure be determined before or after sequencing takes place? Will novel variants be disclosed? What levels of pathogenicity will be disclosed?
Participant choices	Can participants choose whether or not to receive results? Can participants choose specific categories of results? Will participants be asked to use a distinct process to make the decision of whether to receive carrier results?
Results disclosure process	Will results be disclosed via in person meeting, phone, email/letter, or internet? Who will convey the results? Will both positive and negative results be disclosed? Will positive and negative results be conveyed in the same way and/or by the same type of provider?

#### 4.2.1 Selection of genes/conditions to offer for carrier status

Translational genomics research projects should decide whether to offer participants the choice to receive carrier results as a secondary finding from CGES (Darnell et al., 2016). Carrier results for autosomal recessive disorders have thus far not been included on the ACMG list of actionable results that are recommended to be offered to patients in clinical sequencing (Green et al., 2013; Kalia et al., 2017), but in the context of research, investigators may decide to offer carrier results to respond to participants’ interest in these results or to create an evidence base to inform this practice (Jarvik et al., 2014). Research teams should exercise discretion when deciding to offer carrier results and only do this if they have capabilities to provide appropriate handling, interpretation, and communication (Grody, 2016). We are not suggesting that carrier results must be routinely offered as part of CGES research as consensus has not been reached.

Ideally, studies would use specific, predetermined criteria to decide which variants/genes/conditions to disclose. Given the lack of consensus on such criteria or thresholds (Himes et al., 2017; Wilfond & Goddard, 2015), each project should have a deliberative process for developing and applying those criteria. We suggest that projects consider the well‐established association of the gene and variant with a condition as a primary criterion.

Two additional criteria to be considered are the frequency and seriousness of the condition. We acknowledge that frequency and seriousness exist on a continuum, and it is not clear what threshold should be used. An even more challenging caveat is that seriousness, unlike frequency, has a subjective dimension (Wertz & Knoppers, 2002). Seriousness appears to be related to motivations for receiving carrier results, although different individuals will have varying views about what should be considered serious (Schneider et al., 2016). Seriousness can be defined in a range of ways, including categorical features such as effects on life span, cognitive functioning, health system interactions, intensity of symptoms, variability of presentation, and age of onset (Korngiebel et al., 2016). Individuals’ interpretation of these features may be influenced by personal experience, and families and researchers may view them differently. More research is needed to use the “seriousness” criterion optimally. For instance, in one CSER project, an aspect of “seriousness” that was considered important was whether a condition was “medically involved” (Korngiebel et al., 2016), meaning that the condition involved occasional or regular medical evaluations and monitoring and/or home medical interventions, such as may occur in more familiar pediatric conditions like asthma or diabetes as well as many autosomal recessive disorders. For some families, focusing on “medical involvement” may decrease interest in testing if a chronic pediatric disease is perceived as something that can be managed and accepted. Likewise, interest might increase if pediatric chronic illness is perceived by families as something to be avoided.

Acknowledging the lack of consensus in the field as to whether carrier results should be disclosed as secondary findings in research projects using CGES (Green et al., 2012; Yu, Harrell, Jamal, Tabor, & Bamshad, 2014), all the CSER projects that disclosed carrier results included known variants that are classified as pathogenic based on ACMG criteria (Richards et al., 2008). Among the CSER projects, VUSs were not disclosed unless researchers had a question specifically addressing the impact of disclosing VUSs. However, there was variability regarding whether projects disclosed likely pathogenic variants as secondary findings. Notably, the ACMG criteria themselves have been interpreted in divergent ways and the same variant can be classified differently by different laboratories (Amendola et al., 2016).

#### 4.2.2 Participant choices

When projects decide to offer carrier testing results, individuals should be able to choose whether or not to receive carrier results. If disclosure of carrier status is an integral part of the study design and participant choice is not an option, that should be clearly conveyed to participants so they can choose whether or not to enroll in the study (Burke, Trinidad, & Clayton, 2013). Carrier status may be important information for some but unwanted information for others, as the value of the information is based on personal choices for reproductive decision‐making, planning for health of the family, and life stage. There is not yet evidence to conclude that offering different categories of carrier results to select from (e.g., lifespan‐limiting, serious, adult‐onset, unpredictable) is feasible or necessary (Korngiebel et al., 2016), although further research could guide such approaches in the future.

Researchers contemplating carrier results disclosure will need to consider how to structure participant decision‐making, including when to ask participants to decide if they are interested in receiving carrier results. One CSER project asked participants to decide about carrier results analysis at a separate time, subsequent to the disclosure of primary findings. Further research is needed on how best to structure participant decision‐making about carrier status. Individuals will differ in how much value they place on carrier results. Other studies have shown that uptake of carrier screening is lower, for example, when invitations are sent by mail, thereby requiring action on the part of the patient, compared to routinely offering carrier testing results during a primary care visit (Bekker et al., 1993; Henneman et al., 2016). In addition to the example of a second request for analysis, other options could include asking participants to use a decision‐aid to determine their interest in receiving carrier status results; some decision‐aids have been used to improve understanding and promote value‐based decision‐making related to genetic testing in other contexts (Birch et al., 2016). Such approaches may not be feasible in all studies, but targeted approaches may allow for disclosure to the subset of participants most interested in these results.

#### 4.2.3 Results disclosure process

Positive results have been disclosed in person, by phone, letter, or online. There are limited data to guide these processes and future studies should collect data about a range of relevant participant and family outcomes, such as understanding, decisional regret, health behaviors, and satisfaction, in order to guide future protocols. Ensuring there is an avenue to address participant questions is vital. Negative results should be disclosed, especially if patients were asked to make an “opt in” decision for carrier status results. Providing negative results can improve understanding by emphasizing the limitations of the negative results. Adequate communication of negative results requires an appreciation that interpretations may change over time, that not all autosomal recessive diseases will be assessed and that there are other causes of childhood medical conditions and birth defects.

## 5 CONCLUSION

These data show that disclosing carrier results in CGES research has been undertaken and a number of challenges associated with this activity exist. Further translational genomics research on CGES is necessary to guide policy development about carrier results. Such studies will need to determine whether to analyze carrier status results as primary or secondary findings, how to determine the roster of carrier status results to analyze, and how to offer and/or disclose carrier results to participants. The experience of the CSER Consortium provides insights for these future studies. Ideally, data collection from such studies can guide future policy decisions on the clinical integration of genome sequencing for carrier status (Wilfond & Goddard, 2015).

## CONFLICT OF INTEREST

Many authors are clinical service providers and some are employed by laboratories which offer fee‐based clinical sequencing. This employment is noted in the author affiliations. Additional conflicts of interest beyond any author's employment affiliation include: LGB is an uncompensated advisor to the Illumina Corporation, receives royalties from Genentech, Inc, and honoraria from Wiley‐Blackwell; WKC is on the scientific advisory board of Regeneron Genetics Center; LAG is an employee of Eli Lilly and Company, is a cofounder and equity holder of Tango Therapeutics, and an equity holder of Foundation Medicine; RCG receives compensation for speaking or consultation from AIA, GenePeeks, Helix, Illumina, Prudential and Veritas, and is cofounder and advisor to Genome Medical, Inc; GPJ is on the scientific advisory boards of ACT‐X and GRAIL; HLR is on the scientific advisory board of Genome Medical, Inc. The other authors declare no conflict of interest.

## References

[mgg3453-bib-0043] Adam, M. P. , Ardinger, H. H. , Pagon, R. A. , & Wallace, S. E. , (eds.) (2016). GeneReviews (internet). Seattle, WA: University of Washington.

[mgg3453-bib-0001] ACOG Committee on Genetics (2004). ACOG committee opinion. Number 298, August 2004. Prenatal and preconceptional carrier screening for genetic diseases in individuals of Eastern European Jewish descent. Obstetrics and Gynecology, 104, 425–428. 15292027 10.1097/00006250-200408000-00050

[mgg3453-bib-0002] Amendola, L. M. , Jarvik, G. P. , Leo, M. C. , McLaughlin, H. M. , Akkari, Y. , Amaral, M. D. , … Rehm, H. L. (2016). Performance of ACMG‐AMP variant‐interpretation guidelines among nine laboratories in the clinical sequencing exploratory research consortium. American Journal of Human Genetics, 98, 1067–1076. 10.1016/j.ajhg.2016.03.024 27181684 PMC4908185

[mgg3453-bib-0003] American College of Obstetricians and Gynecologists Committee on Genetics . (2011). ACOG Committee Opinion No. 486: Update on carrier screening for cystic fibrosis. Obstetrics and Gynecology, 117, 1028–1031. 21422883 10.1097/AOG.0b013e31821922c2

[mgg3453-bib-0004] Bekker, H. , Modell, M. , Denniss, G. , Silver, A. , Mathew, C. , Bobrow, M. , & Marteau, T. (1993). Uptake of cystic fibrosis testing in primary care: Supply push or demand pull? BMJ, 306, 1584–1586. 10.1136/bmj.306.6892.1584 8329922 PMC1678014

[mgg3453-bib-0005] Biesecker, L. G. , & Green, R. C. (2014). Diagnostic clinical genome and exome sequencing. New England Journal of Medicine, 370, 2418–2425. 10.1056/NEJMra1312543 24941179

[mgg3453-bib-0006] Biesecker, B. B. , Lewis, K. L. , Umstead, K. L. , Johnston, J. J. , Turbitt, E. , Fishler, K. P. , … Biesecker, L. G. (2018). Web platform vs in‐person genetic counselor for return of carrier results from exome sequencing: A randomized clinical trial. JAMA Internal Medicine., 178, 338–346. 10.1001/jamainternmed.2017.8049 29356820 PMC5885925

[mgg3453-bib-0007] Birch, P. , Adam, S. , Bansback, N. , Coe, R. R. , Hicklin, J. , Lehman, A. , … Friedman, J. M. (2016). DECIDE: A decision support tool to facilitate parents’ choices regarding genome‐wide sequencing. Journal of Genetic Counseling, 25, 1298–1308. 10.1007/s10897-016-9971-8 27211035

[mgg3453-bib-0008] Burke, W. , Trinidad, S. B. , & Clayton, E. W. (2013). Seeking genomic knowledge: The case for clinical restraint. The Hastings Law Journal, 64, 1650–1664. 24688162 PMC3969739

[mgg3453-bib-0009] Cirino, A. L. , Lakdawala, N. K. , McDonough, B. , Conner, L. , Adler, D. , Weinfeld, M. ,… Ho, C. Y. (2017). A comparison of whole genome sequencing to multigene panel testing in hypertrophic cardiomyopathy patients. Circulation: Cardiovascular Genetics, 10, e001768 10.1161/CIRCGENETICS.117.001768 29030401 PMC5683423

[mgg3453-bib-0010] Darnell, A. J. , Austin, H. , Bluemke, D. A. , Cannon, R. O. 3rd , Fischbeck, K. , Gahl, W. ,… Biesecker, L. G. (2016). A clinical service to support the return of secondary genomic findings in human research. American Journal of Human Genetics, 98, 435–441. 10.1016/j.ajhg.2016.01.010 26942283 PMC4800041

[mgg3453-bib-0011] Edwards, J. G. , Feldman, G. , Goldberg, J. , Gregg, A. R. , Norton, M. E. , Rose, N. C. , … Watson, M. S. (2015). Expanded carrier screening in reproductive medicine‐points to consider: A joint statement of the American College of Medical Genetics and Genomics, American College of Obstetricians and Gynecologists, National Society of Genetic Counselors, Perinatal Quality Foundation, and Society for Maternal‐Fetal Medicine. Obstetrics and Gynecology, 125, 653–662. 10.1097/AOG.0000000000000666 25730230

[mgg3453-bib-0012] Fabsitz, R. R. , McGuire, A. , Sharp, R. R. , Puggal, M. , Beskow, L. M. , Biesecker, L. G. ,… Burke , G. L. (2010). Ethical and practical guidelines for reporting genetic research results to study participants: Updated guidelines from a National Heart, Lung, and Blood Institute working group. Circulation: Cardiovascular Genetics, 3, 574–580. 10.1161/CIRCGENETICS.110.958827 21156933 PMC3090664

[mgg3453-bib-0013] Green, R. C. , Berg, J. S. , Berry, G. T. , Biesecker, L. G. , Dimmock, D. P. , Evans, J. P. ,… Jacob , H. J. (2012). Exploring concordance and discordance for return of incidental findings from clinical sequencing. Genetics in Medicine, 14, 405–410. 10.1038/gim.2012.21 22422049 PMC3763716

[mgg3453-bib-0014] Green, R. C. , Berg, J. S. , Grody, W. W. , Kalia, S. S. , Korf, B. R. , Martin, C. L. ,… Biesecker, L. G. (2013). ACMG recommendations for reporting of incidental findings in clinical exome and genome sequencing. Genetics in Medicine, 15, 565–574. 10.1038/gim.2013.73 23788249 PMC3727274

[mgg3453-bib-0015] Green, R. C. , Goddard, K. A. , Amendola, L. M. , Appelbaum, P. S. , Berg, J. S. , Bernhardt, B. A. ,… CSER Consortium . (2016). The clinical sequencing exploratory research consortium: Accelerating the evidence‐based practice of genomic medicine. American Journal of Human Genetics, 98, 1051–1066. 10.1016/j.ajhg.2016.04.011 27181682 PMC4908179

[mgg3453-bib-0016] Grody, W. W. (2016). Where to draw the boundaries for prenatal carrier screening. JAMA, 316, 717–719. 10.1001/jama.2016.10888 27533155

[mgg3453-bib-0017] Grody, W. W. , Thompson, B. H. , Gregg, A. R. , Bean, L. H. , Monaghan, K. G. , Schneider, A. , & Lebo, R. V. (2013). ACMG position statement on prenatal/preconception expanded carrier screening. Genetics in Medicine, 15, 482–483. 10.1038/gim.2013.47 23619275

[mgg3453-bib-0018] Henneman, L. , Borry, P. , Chokoshvili, D. , Cornel, M. C. , van El, C. G. , Forzano, F. ,… Peterlin, B. (2016). Responsible implementation of expanded carrier screening. European Journal of Human Genetics, 24, e1–e12. 10.1038/ejhg.2015.271 PMC4867464 26980105

[mgg3453-bib-0019] Himes, P. , Kauffman, T. L. , Muessig, K. R. , Amendola, L. , Berg, J. S. , Dorschner, M. O. ,… Goddard, K. A. B. (2017). Genome sequencing and carrier testing: Decisions on categorization and whether to disclose results of carrier testing. Genetics in Medicine., 19, 803–808. 10.1038/gim.2016.198 28079899 PMC5509491

[mgg3453-bib-0020] Ioannou, L. , McClaren, B. J. , Massie, J. , Lewis, S. , Metcalfe, S. A. , Forrest, L. , & Delatycki, M. B. (2014). Population‐based carrier screening for cystic fibrosis: A systematic review of 23 years of research. Genetics in Medicine, 16, 207–216. 10.1038/gim.2013.125 24030436

[mgg3453-bib-0021] Jarvik, G. P. , Amendola, L. M. , Berg, J. S. , Brothers, K. , Clayton, E. W. , Chung, W. ,… Burke, W. (2014). Return of genomic results to research participants: The floor, the ceiling, and the choices in between. American Journal of Human Genetics, 94, 818–826. 10.1016/j.ajhg.2014.04.009 24814192 PMC4121476

[mgg3453-bib-0022] Kalia, S. S. , Adelman, K. , Bale, S. J. , Chung, W. K. , Eng, C. , Evans, J. P. ,… Miller, D. T. (2017). Recommendations for reporting of secondary findings in clinical exome and genome sequencing, 2016 update (ACMG SF v2.0): A policy statement of the American College of Medical Genetics and Genomics. Genetics in Medicine, 19, 249–255. 10.1038/gim.2016.190 27854360

[mgg3453-bib-0023] Kauffman, T. L. , Wilfond, B. S. , Jarvik, G. P. , Leo, M. C. , Lynch, F. L. , Reiss, J. A. ,… Goddard, K. A. (2017). Design of a randomized controlled trial for genomic carrier screening in healthy patients seeking preconception genetic testing. Contemporary Clinical Trials, 53, 100–105. 10.1016/j.cct.2016.12.007 27940182 PMC5274557

[mgg3453-bib-0024] Korngiebel, D. M. , McMullen, C. K. , Amendola, L. M. , Berg, J. S. , Davis, J. V. , Gilmore, M. J. ,… Wilfond, B. S. (2016). Generating a taxonomy for genetic conditions relevant to reproductive planning. American Journal of Medical Genetics, 170, 565–573. 10.1002/ajmg.a.37513 26889673 PMC4860293

[mgg3453-bib-0025] Lazarin, G. A. , Haque, I. S. , Nazareth, S. , Iori, K. , Patterson, A. S. , Jacobson, J. L. ,… Srinivasan, B. S. (2013). An empirical estimate of carrier frequencies for 400 + causal Mendelian variants: Results from an ethnically diverse clinical sample of 23,453 individuals. Genetics in Medicine, 15, 178–186. 10.1038/gim.2012.114 22975760 PMC3908551

[mgg3453-bib-0026] Lewis, K. L. , Umstead, K. L. , Johnston, J. J. , Miller, I. M. , Thompson, L. J. , Fishler, K. P. ,… Biesecker, B. B. (2018). Outcomes of counseling after education about carrier results: A randomized controlled trial. American Journal of Human Genetics, 102(4), 540–546. 29526281 10.1016/j.ajhg.2018.02.009 PMC5985358

[mgg3453-bib-0027] Nazareth, S. B. , Lazarin, G. A. , & Goldberg, J. D. (2015). Changing trends in carrier screening for genetic disease in the United States. Prenatal Diagnosis, 35, 931–935. 10.1002/pd.4647 26138560 PMC4758394

[mgg3453-bib-0028] Parens, E. , & Asch, A. (1999). The disability rights critique of prenatal genetic testing. Reflections and recommendations. Hastings Center Report, 29, S1–S22. 10.2307/3527746 10587809

[mgg3453-bib-0042] Parens, E. & Asch, A. , (eds.) (2000). Prenatal testing and disability rights. Washington, DC: Georgetown University Press.

[mgg3453-bib-0029] Parsons, D. W. , Roy, A. , Yang, Y. , Wang, T. , Scollon, S. , Bergstrom, K. ,… Plon, S. E. (2016). Diagnostic yield of clinical tumor and germline whole‐exome sequencing for children with solid tumors. JAMA Oncology, 10.1001/jamaoncol.2015.5699. [Epub ahead of print]. PMC5471125 26822237

[mgg3453-bib-0030] Presidential Commission for the Study of Bioethical Issues . (2013). Anticipate and Communicate: Ethical Management of Incidental and Secondary Findings in the Clinical, Research, and Direct‐to‐Consumer Contexts. (December 2013 Report of the Presidential Commission for the Study of Bioethical Issues) (Washington, DC: Presidential Commission for the Study of Bioethical Issues). 10.1093/aje/kwu217 25150271

[mgg3453-bib-0031] Press, N. , Wilfond, B. S. , Murray, M. , & Burke, W. (2011). The power of knowledge: How carrier and prenatal screening altered the clinical goals of genetic testing In Burke W., Edwards K., Goering S., Holland S., & Trinidad S. B. (Eds.), Achieving justice in genomic translation (pp. 95–107). New York, NY: Oxford University Press.

[mgg3453-bib-0032] Ravitsky, V. , & Wilfond, B. S. (2006). Disclosing individual genetic results to research participants. American Journal of Bioethics, 6, 8–17. 10.1080/15265160600934772 17085395

[mgg3453-bib-0033] Richards, C. S. , Bale, S. , Bellissimo, D. B. , Das, S. , Grody, W. W. , Hegde, M. R. , … Ward, B. E. (2008). ACMG recommendations for standards for interpretation and reporting of sequence variations: Revisions 2007. Genetics in Medicine, 10, 294–300. 10.1097/GIM.0b013e31816b5cae 18414213

[mgg3453-bib-0034] Richardson, H. S. , & Belsky, L. (2004). The ancillary‐care responsibilities of medical researchers: An ethical framework for thinking about the clinical care that researchers owe their subjects. Hastings Center Report, 34, 25–33. 10.2307/3528248 15098404

[mgg3453-bib-0035] Schneider, J. L. , Goddard, K. A. , Davis, J. , Wilfond, B. , Kauffman, T. L. , Reiss, J. A. ,… McMillen, C. (2016). “is it worth knowing?” Focus group participants’ perceived utility of genomic preconception carrier screening. Journal of Genetic Counseling, 25, 135–145. 10.1007/s10897-015-9851-7 26093606 PMC4726717

[mgg3453-bib-0036] Vassy, J. L. , Christensen, K. D. , Schonman, E. F. , Blout, C. L. , Robinson, J. O. , Krier, J. B. ,… MedSeg Project . (2017). The impact of whole‐genome sequencing on the primary care and outcomes of healthy adult patients: A pilot randomized trial. Annals of Internal Medicine, [Epub ahead of print]. 10.1016/j.ajhg.2014.06.004 PMC5856654 28654958

[mgg3453-bib-0037] Wald, N. J. , George, L. M. , Wald, N. M. , & Mackenzie, I. (1993). Couple screening for cystic fibrosis. Lancet, 342, 1307–1308. 10.1016/0140-6736(93)92403-G 7901621

[mgg3453-bib-0038] Wertz, D. C. , & Knoppers, B. M. (2002). Serious genetic disorders: can or should they be defined? American Journal of Medical Genetics, 108, 29–35. 10.1002/ajmg.10212 11857546

[mgg3453-bib-0039] Wilfond, B. , & Goddard, K. A. B. (2015). It's complicated: Criteria for policy decisions for the clinical integration of genome scale sequencing for reproductive decision‐making. Molecular Genetics and Genomic Medicine, 3, 239–242. 10.1002/mgg3.130 26247041 PMC4521960

[mgg3453-bib-0040] Wolf, S. M. , Crock, B. N. , Van Ness, B. , Lawrenz, F. , Kahn, J. P. , Beskow, L. M. ,… Wolf, W. A. (2012). Managing incidental findings and research results in genomic research involving biobanks and archived data sets. Genetics in Medicine, 14, 361–384. 10.1038/gim.2012.23 22436882 PMC3597341

[mgg3453-bib-0041] Wynn, J. , Martinez, J. , Bulafka, J. , Duong, J. , Zhang, Y. , Chiuzan, C. ,… Chung, W. K. (2018). Impact of receiving secondary results from genomic research: A 12‐month longitudinal study. Journal of Genetic Counseling, 27(3), 709–722. 29168042 10.1007/s10897-017-0172-x PMC5945295

[mgg3453-bib-0044] Yu, J. H. , Harrell, T. M. , Jamal, S. M. , Tabor, H. K. , & Bamshad, M. J. (2014). Attitudes of genetics professionals toward the return of incidental results from exome and whole‐genome sequencing. American Journal of Human Genetics, 95, 77–84. 24975944 10.1016/j.ajhg.2014.06.004 PMC4085580

